# Genomic Selection for growth traits in *Eucalyptus*: accuracy within and across breeding populations

**DOI:** 10.1186/1753-6561-5-S7-O16

**Published:** 2011-09-13

**Authors:** Dario Grattapaglia, Marcos Deon Vilela Resende, Márcio Ribeiro Resende, Carolina Paola Sansaloni, Cesar Daniel Petroli, Alexandre Alves Missiaggia, Elisabete Keiko Takahashi, Karina Carnielli Zamprogno, Andrzej Kilian

**Affiliations:** 1 EMBRAPA Genetic Resources and Biotechnology – Estação Parque Biológico, 70770-910, Brazilia, DF, Brazil; 2 EMBRAPA Forestry Research, Colombo, PR, 83411-000, Brazil; 3 Universidade Federal de Viçosa – Bioagro, Viçosa MG, 36570-000, Brazil; 4 Universidade de Brazilia – Campus Darcy Ribeiro Brasília, DF, 70910-900, Brazil; 5 FIBRIA, Rod. Aracruz/Barra do Riacho, km 25, Aracruz, ES, 29197-900, Brazil; 6 CENIBRA Celulose Nipo Brazileira S.A, Belo Oriente, MG, 35196-000, Brazil; 7 VERACEL Celulose S.A., Eunápolis, BA, 45820-970, Brazil; 8 DArT - Diversity Arrays Technology, POB 7141, Yarralumla, ACT, 2600, Australia

## Background

Genomic selection (GS) involves selection decisions based on genomic breeding values estimated as the sum of the effects of genome-wide markers capturing most QTLs for the target trait(s). GS is revolutionizing breeding practice for complex trait in domestic animals. The same approach and concepts can be readily applied to forest tree breeding. Trees also have long generation times and late expressing traits. Differently from association genetics that aims at dissecting complex traits in their discrete components, GS precludes the discovery of individual marker-trait associations and focuses on prediction of performance. By capturing the "missing heritability" of complex quantitative traits beyond the few effect variants that association genetics has so far typically identified, GS might soon cause a paradigm shift in forest tree breeding. In a prior deterministic study we assessed the impact of linkage disequilibrium (modeled by Ne and inter-marker distance), the size of the training set, trait heritability and the number of QTL on the predicted accuracy of GS [1]. Results indicate that GS has the potential to radically improve the efficiency of tree breeding. The benchmark accuracy of conventional BLUP-based phenotypic selection (0.68) was reached by GS even at a marker density ~2 markers/cM when Ne ≤30, while up to 10 markers/cM are necessary for larger Ne. Shortening the breeding cycle by 50% with GS provides an expected increase ≥100% in selection efficiency. To validate these simulation results we carried out a large multi-population proof-of-concept study of GS in tropical Eucalyptus. In this report we present results of this on-going study for two populations and three different quantitative traits.

## Methods

This study was carried out in two structured populations of Eucalyptus represented by two progeny trials of independent breeding programs. The first was a progeny trial developed by CENIBRA (CEN) with 43 full-sib families generated by an incomplete diallel mating design carried out by intercrossing 11 highly selected *E. grandis x E. urophylla* hybrids clones therefore having an effective population size of Ne= 11. The second population was a progeny trial installed by FIBRIA (FIB) with 232 full-sib families involving 120 elite parents, the vast majority F1 hybrids of *E. grandis*, *E. urophylla*, *E. globulus* and* E. maidenii*. For the GS study however only the most phenotypically relevant 75 families were used derived from intercrossing 55 parents therefore limiting the effective population size to Ne= 55. A sample of trees was taken in a stratified way from all families to provide a balanced representation of all families with similar numbers of individuals per family. Bark or leaf samples were collected, DNA extracted and genotypes obtained for 783 and 920 trees from CEN and FIB respectively. Genotyping was carried out using a high-throughput genotyping platform developed earlier [2]that provided 3,120 and 3,564 robustly scored dominant DArT markers. De-regressed phenotypes for Height (H), Diameter at Breast Height (DBH), wood density (WD), pulp yield, lignin content and *Puccinia* rust resistance were obtained. Single marker regression association analyses were initially performed with all the traits, treating the markers as fixed effects. Markers selected in the previous association analysis had their effects estimated adjusting all the allelic effects simultaneously using Random Regression Best Linear Unbiased Predictor. Each population was then divided in an training set comprising 90% of the total number of individuals and a validation set with the other 10%. The estimated effects of the markers were validated using a cross validation approach using random sub-sampling with ten replications so that all individuals had their phenotypes predicted by GS. Details of the overall analytical approach have been described [3,4].

## Results and conclusions

To date results have been obtained for height, DBH and wood density while the remaining traits are under analysis. Realized accuracies by Genomic Estimated Breeding Values (GEBV) for H and DBH were 0.67 and 0.69 for CEN and 0.62 and 0.54 for FIB while for WD in FIB it was 0.53. These accuracies match or surpass those obtained by conventional BLUP (Best Linear Unbiased Prediction) based phenotypic selection. Not surprising GEBV accuracies were low (~0.18) across populations implying variable genotype-phenotype associations across backgrounds so that population-specific GS models will be necessary. GS-based reduction in breeding time by 50%, i.e. by reducing a breeding generation time from 12 to 6 years should provide gains ≥100% in selection efficiencies. If a reduction from 12 to 3 years using flower induction can be achieved, gains in selection efficiency theoretically could surpass 300%. These are among the first experimental results of GS in forest trees and in plants in general. With the technological advances and declining costs of genotyping methods we anticipate that GS will soon be implemented operationally and revolutionize *Eucalyptus* tree breeding practice.
